# Atrial fibrillation during palliative chemotherapy with utidelone: a case report

**DOI:** 10.3389/fonc.2026.1695712

**Published:** 2026-04-29

**Authors:** Qihuan Shen, Yuhao Huang, Haoran Li, Baoying Chen, Huadi Shi, Zumin Xu

**Affiliations:** Department of Cancer Center, Affiliated Hospital of Guangdong Medical University, Zhanjiang, Guangdong, China

**Keywords:** adverse drug reaction, atrial fibrillation, atrial flutter, breast cancer, utidelone

## Abstract

This report describes a 57-year-old male patient with metastatic breast cancer who experienced atrial fibrillation during the infusion of utidelone as palliative chemotherapy. All cardiological examinations conducted before treatment were normal. There was no evidence of thyroid dysfunction, nor any objective data indicating acute or chronic cardiovascular disease. Other combination drugs have been used in clinical practice for decades and rarely have proarrhythmic activity. The innovative drug utidelone, independently developed in China, was approved by the National Medical Products Administration (NMPA) of China in 2021 for the treatment of patients with metastatic breast cancer. Cardiotoxicity has not been prominently reported in prior clinical trials of utidelone. Although a definite causal relationship cannot be confirmed, the close temporal association and clinical course suggest that utidelone may have contributed to the arrhythmias. This case highlights the need for careful cardiac monitoring during treatment.

## Introduction

Breast cancer is the most common tumor in women and a main cause of death among women (1). Triple negative breast cancer (TNBC) is a subtype of breast cancer, accounting for approximately 10% to 20% of all breast cancers. Compared with other types of breast cancer, patients with TNBC have a higher incidence of distant metastases. They cannot benefit from conventional endocrine therapy and anti-HER2 targeted therapy, and the main treatment method is chemotherapy (2). The main chemotherapy drugs for advanced triple-negative breast cancer include doxorubicin, paclitaxel, eribulin, platinum, and utidelone (UTD1), etc. Among them, UTD1 has demonstrated favorable efficacy, tolerability, and manageable safety in patients with advanced anthracycline/taxane refractory metastatic breast cancer (3, 4). Common adverse effects of UTD1 include peripheral neuropathy, musculoskeletal pain, fatigue, nausea, neutropenia, leukopenia, diarrhea, and anorexia (5). Currently, there are no literature records of atrial fibrillation caused by UTD1. Compared with female breast cancer, male breast cancer (MBC), although rare, also faces severe challenges. Male breast cancer accounts for only about 1% of all breast cancers, and the triple-negative subtype is even rarer. Moreover, due to the lack of large-scale prospective clinical trials, its treatment strategies often refer to the guidelines for female patients (6). Here, we report a case of atrial fibrillation and atrial flutter in a patient with metastatic breast cancer after receiving UTD1 treatment.

## Case presentation

A 57-year-old male presented with a right axillary mass and underwent palliative resection of the right axillary mass and segmental mastectomy on August 31, 2021. Postoperative pathology revealed adenocarcinoma with neuroendocrine differentiation. Immunohistochemistry (IHC) results: Estrogen receptor (ER) (–), progesterone receptor (PR) (–), HER2 (2+, FISH negative), Ki-67 (40%+). Postoperative diagnosis was occult breast cancer with axillary lymph node metastasis (pT0N1M0, Stage IIA, TNBC). Adjuvant chemotherapy (4 cycles of doxorubicin plus cyclophosphamide, followed by 4 cycles of docetaxel) was administered from September 2021 to February 2022. This was followed by adjuvant radiotherapy and subsequent adjuvant intensification therapy with capecitabine, which the patient self-discontinued after two months. A magnetic resonance imaging (MRI) scan on December 12, 2023, revealed a left temporal lobe metastatic lesion. The patient underwent local resection of the left temporal lobe tumor on December 19, 2023. Pathology confirmed metastatic poorly differentiated adenocarcinoma with neuroendocrine differentiation. Efficacy evaluation indicated progressive disease, with a disease-free survival (DFS) of 26 months.

**Figure 1:**
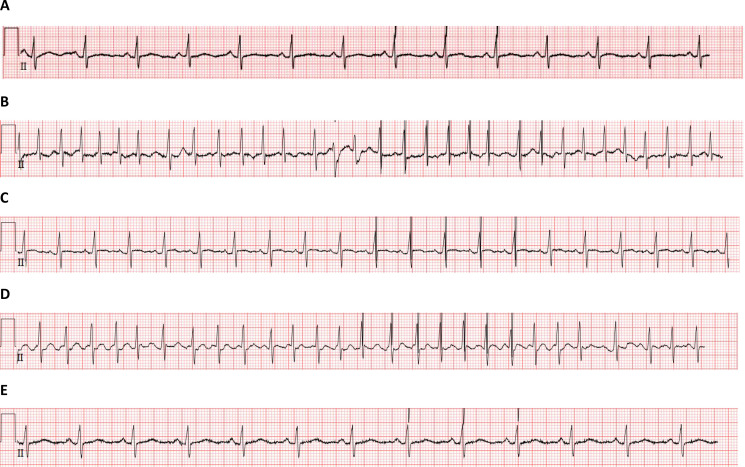
The ECG changes of patients before and after medication. **(A)** The ECG before medication on January 22, 2024. **(B)** The ECG after medication on January 29, 2024 at 10:31 AM showing Atrial Fibrillation. **(C)** The ECG after medication on January 29, 2024 at 4:03 PM showing sinus rhythm. **(D)** The ECG after medication on January 31, 2024 at 8:53 PM showing atrial flutter. **(E)** The ECG after medication on June 25, 2024 showing sinus rhythm.

During Cycle 1 of first-line therapy for metastatic disease, the patient received bevacizumab (BEV) plus utidelone (UTD1). About 24 hours after the last infusion of utidelone ended, he developed sudden-onset arrhythmia. The detailed progression of the disease was as follows (**Table 1**): Admitted on January 22, 2024, the patient denied chest tightness, pain, palpitations, or dyspnea. His mental status, appetite, sleep, and bowel/bladder function were unremarkable. His medical history was negative for coronary artery disease, arrhythmias, hypertension, hyperthyroidism, or diabetes mellitus. Physical examination findings: temperature 36.3 °C, pulse 85 beats per minute (bpm), respiratory rate 18 breaths per minute, blood pressure 148/102 mmHg. Heart rate was 85 bpm with regular rhythm and no audible cardiac murmurs. Initial laboratory investigations—including cardiac enzymes, electrolytes, liver function, and renal function—were within normal limits. Baseline electrocardiogram (ECG) (**Figure 1A**) and echocardiography indicated an ejection fraction of 60% (**Table 2**). From January 24 to January 28, 2024, the patient received BEV 400 mg on day 1 (D1) and UTD1–50 mg on days 1–5 (D 1–5). Premedication included dexamethasone sodium phosphate, ranitidine, dolasetron mesylate, and fosaprepitant dimeglumine.

**Table 1:** Timeline of events.

Date	Clinical event
Aug 31, 2021	Right axillary mass resection and segmental mastectomy
Sep 2021 to Feb 2022	Adjuvant chemotherapy
Mar 2022	Adjuvant radiotherapy
Apr 2022 to Jun 2022	Adjuvant chemotherapy with capecitabin
Dec 12, 2023	Brain MRI showed metastatic lesion
Dec 19, 2023	Resection of metastatic brain lesion
Jan 22, 2024	Normal baseline ECG and echocardiography
Jan 24 to 28, 2024	BEV 400 mg D1 + UTD1–50 mg D1–5
Jan 29, 2024 at 10:30 AM	Palpitations/chest tightness; ECG showed AF
Jan 29, 2024 at 4:03 PM	Antiarrhythmic treatment resumed; rhythm controlled
Jan 31, 2024 at 9:09 PM	Recurrent symptoms; ECG showed AFL.
Jun 25, 2024	Follow-up ECG showed sinus rhythm.

**Table 2:** Echocardiogram results.

Date	Left ventricular ejection fraction EF (%)
December 18, 2023	60
December 31, 2023	56
April 16, 2024	58

On January 29, 2024, at 10:30 AM, the patient felt palpitations and chest tightness. Examination revealed a pulse rate of 98 bpm, oxygen saturation of 99% by pulse oximetry, and an auscultated heart rate of approximately 135 bpm with an irregularly irregular rhythm and variable intensity of the first heart sound. Cardiac monitoring was initiated immediately. The ECG (**Figure 1B**) demonstrated atrial fibrillation (AF) with rapid ventricular response. Laboratory results showed elevated pro-brain natriuretic peptide (Pro-BNP) at 1152.0 pg/mL and normal myoglobin at 33.49 ng/mL. Immediate management comprised intravenous amiodarone 150 mg and deslanoside 0.2 mg for ventricular rate control, followed by oral rivaroxaban for anticoagulation. By 12:26 AM, the chest tightness had resolved, and the monitored heart rate was 105 bpm. A repeat ECG at 4:54 PM (**Figure 1C**) confirmed restoration of sinus rhythm. Due to persistent tachycardia (approximately 121 bpm), oral metoprolol was initiated for rate control. A recheck of Pro-BNP (877.0 pg/mL) showed improvement. Echocardiography on January 31, 2024, revealed normal cardiac structure, coordinated wall motion, no significant valvular abnormalities and an ejection fraction of 56%(**Table 2**). However, at 9:09 PM that same day, the patient experienced recurrent and worsening palpitations and chest tightness. An urgent ECG (**Figure 1D**) showed AFL with a heart rate of 170 bpm. Treatment was restarted with an intravenous infusion of deslanoside 0.2 mg and amiodarone 300 mg. The heart rate subsequently decreased to 110–120 bpm, and sinus rhythm was restored. Given the efficacy of this antiarrhythmic regimen, maintenance therapy with oral amiodarone and metoprolol was continued. Due to these severe adverse events, the BEV plus UTD1 regimen was permanently discontinued. A follow-up ECG (**Figure 1E**) obtained six months after medication discontinuation demonstrated no recurrence of AF or AFL.

## Discussion

This case describes the occurrence of atrial fibrillation and atrial flutter during BEV plus UTD1 therapy in a male patient with metastatic triple-negative breast cancer.

The patient had no prior history of cardiac disease, arrhythmias or hyperthyroidism. A baseline ECG obtained within 24 hours of admission confirmed sinus rhythm (**Figure 1A**). Notably, within 24 hours and 72 hours following completion of the first cycle of UTD1 therapy, he developed two distinct, rare, and severe cardiotoxic events, namely AF with rapid ventricular response (**Figure 1B**) and AFL (**Figure 1D**). These events occurred shortly after completion of UTD1 administration, suggesting a temporal association, although this timing alone is insufficient to establish causality. Both resolved with aggressive intervention, and no recurrence was observed following discontinuation of UTD1 containing therapy. The absence of subsequent UTD1 exposure due to treatment discontinuation precludes observation of a rechallenge effect, limiting the ability to provide more robust evidence strengthening the association between UTD1 and AF. Importantly, BEV, having been on the market for more than two decades, has clearly defined common cardiovascular adverse reactions, including hypertension and arterial thromboembolic events (7, 8). However, neither its drug label nor a large amount of post-marketing surveillance data and review literature list it as a definite risk factor for AF or AFL (9, 10). Premedication with dolasetron mesylate and fosaprepitant dimeglumine was administered without preceding arrhythmic symptoms. Application of the Naranjo algorithm adverse drug reaction (ADR) probability scale yielded a score of 5 (11, 12), suggesting UTD1 may have contributed to the development of atrial fibrillation in this patient (**Table 3**). However, this result should be interpreted cautiously given the presence of concomitant therapy and competing clinical explanations.

**Table 3:** Naranjo adverse drug reaction probability scale.

Related Issues	Yes	No	Unknown	Score
1. Are there previous conclusive reports on this reaction?	+1	0	0	0
2. Did the adverse event appear after the suspected drug was administered?	+2	-1	0	2
3. Did the adverse reaction improve when the drug was discontinued or when a specific antagonist was given?	+1	0	0	1
4. Did the adverse reaction reappear when the drug was readministered?	+2	-1	0	0
5. Are there alternative causes that could solely have caused the reaction?	-1	+2	0	2
6. Did the reaction reappear when a placebo was given?	-1	-1	0	0
7. Was the drug detected in the blood at toxic concentrations?	+1	0	0	0
8. Was the reaction more severe when the dose was increased or less severe when the dose was decreased?	+1	0	0	0
9. Did the patient have a similar reaction to the same/similar drugs in any previous exposure?	+1	0	0	0
10. Was the adverse event confirmed by any objective evidence?	+1	0	0	0

Total Score: 5 Interpretation: Probable.

<0:Doubtful; 1-4: Possible; 5-8: Probable; ≥9: Definite.

We must recognize that there are significant sex differences in the epidemiology of atrial fibrillation. Men have a higher risk of developing AF. The 2021 Global Burden of Disease Study found age-standardized prevalence rates of 728.88 per 100,000 in men versus 529.12 per 100,000 in women (13). In addition, this case is a rare male patient with triple-negative breast cancer. Male breast cancer patients are often excluded from large-scale clinical trials, and there may be gender differences in the spectrum of adverse drug reactions. In the early stage of the key clinical trial of UTD1, male subjects were not included. Therefore, it is currently unclear whether there are gender-dependent differences in pharmacokinetics or cardiotoxicity of this drug. This case suggests that gender subgroup analysis should be included in future drug safety studies.

However, emerging evidence in cardio-oncology suggests that drug-induced cardiac electrophysiological toxicity may not align with immediate plasma peak concentrations but may instead involve cumulative effects in cardiac tissue, sustained impacts on cellular structures, or the triggering of subsequent inflammatory or oxidative stress cascades (14, 15). For instance, anthracycline-induced ventricular arrhythmias have been reported to occur 23 days after chemotherapy initiation, with persistence for up to one month after drug cessation. Similarly, fluconazole-associated torsades de pointes manifested after 14 days of continuous administration and resolved only upon drug withdrawal. Even in cases of acute cardiotoxicity, recovery may take months. These examples support the notion that drug-induced arrhythmias do not necessarily align with immediate drug exposure but may follow a delayed and sustained course. In this case, the patient developed atrial fibrillation on January 29th. After cardioversion, atrial flutter (AFL) recurred approximately 72 hours after the last dose of UTD1, suggesting that the state of cardiac electrical instability persisted for several days after drug withdrawal. This pattern of “occurrence and persistence after drug withdrawal” is consistent with the characteristic that drug-induced arrhythmia is not solely determined by the immediate blood drug concentration, supporting the possibility of UTD1 being a inducing factor.

Malignancies, particularly metastatic tumors, can increase the risk of AF through systemic inflammatory states, cytokine release, hypercoagulability, and paraneoplastic phenomena (16, 17). The patient diagnosed with metastatic triple-negative breast cancer with brain metastases in this case, indeed possessed this underlying risk profile. Furthermore, pathology of the brain metastases revealed neuroendocrine differentiation. As documented in the literature, tumors exhibiting neuroendocrine differentiation may secrete various vasoactive substances such as 5-hydroxytryptamine and plasmakinin and other bioactive peptides, which could theoretically impact the cardiovascular system (18). However, the patient did not present with clinical manifestations of carcinoid syndrome, such as flushing or diarrhea, suggesting the absence of significantly active hormone secretion. Critically, the brain metastases were surgically resected in December 2023, and a baseline ECG prior to chemotherapy administration was normal. This observation partially diminishes the likelihood that an active tumor secretory state served as the primary cause of the acute atrial fibrillation episode. We propose that the oncological condition may represent the “soil”, whereas the acute administration of UTD1 may have acted as the “seed” for the arrhythmic event.

UTD1 is, in essence, a derivative of epothilone B. Its mechanism of action bears similarities to those of paclitaxel and epothilone. As a microtubule stabilizer, utidelone binds to β-tubulin with high affinity. This binding stabilizes the microtubule structure, and inhibits microtubule depolymerization and dynamic instability. Consequently, it leads to mitotic arrest and ultimately induces apoptosis in tumor cells. Due to its capacity to overcome multidrug resistance mediated by efflux transporters such as P-glycoprotein (P-gp), it is commonly employed as a second-line therapeutic option following the development of paclitaxel resistance in breast cancer.

The mechanism by which UTD1 induces malignant arrhythmia remains unclear, and no prior literature reports this specific adverse event. Data concerning the cardiovascular toxicity profile of UTD1 are lacking. However, Zhang et al. previously reported AF associated with paclitaxel therapy in breast cancer patients, proposing potential mechanisms including autonomic dysfunction, cardiac structural remodeling, ion channel dysregulation, electrical remodeling, and blood pressure abnormalities (19). Given the structural similarity between paclitaxel and UTD1, it is plausible that UTD1 may induce AF via analogous pathways.

Furthermore, there is a paucity of clinical studies examining the adverse effects associated with UTD1-related arrhythmias; thus, a direct causal relationship between the two cannot be established. Nevertheless, this serendipitous finding suggests that utidelone may pose a risk for inducing significant arrhythmias. Consequently, heightened vigilance and intensified cardiac monitoring are warranted during its clinical use.

Advances in multimodal breast cancer therapy have significantly improved survival. However, chemotherapy-associated cardiovascular toxicity has emerged as a major factor limiting long-term outcomes (20). Studies indicate that breast cancer patients receiving chemotherapy face an increased risk of arrhythmias compared to the general population, exhibiting time-dependency (21). Therefore, prevention and early detection of severe malignant arrhythmias during anti-tumor therapy are paramount. The emergence of a malignant arrhythmia such as AF requires the immediate cessation of potentially causative agents and the prompt initiation of therapy to restore sinus rhythm and control ventricular rate. Concurrently, comprehensive investigations for myocardial injury and continuous ECG monitoring are essential. Upon stabilization, the treatment regimen should be reassessed and potentially modified based on individual patient risk factors. Furthermore, longitudinal surveillance of cardiac function remains crucial even after completion of anti-tumor therapy to enable timely intervention and prevent or mitigate the progression of cardiac dysfunction (22).

This report has several limitations. First, it describes a single patient and therefore cannot establish causality. Second, the patient received combination therapy, making it difficult to isolate the effect of UTD1 from that of bevacizumab or other clinical variables. Third, the delayed timing of arrhythmia onset relative to drug administration complicates causal interpretation. Fourth, the patient’s metastatic disease status and neuroendocrine differentiation represent additional potential confounding factors. Finally, no rechallenge was performed. Therefore, this case should be interpreted as a signal-generating observation that warrants further pharmacovigilance and clinical investigation.

## Conclusion

Based on the clinical presentation, patient history, and adverse drug reaction assessment (Naranjo score 5), we consider UTD1 a possible contributing factor to AF in this case, while acknowledging that causality remains unproven. To our knowledge, this represents the first documented case of both atrial fibrillation and atrial flutter occurring following utidelone administration. This case underscores the imperative for enhanced vigilance and proactive cardiac monitoring when utilizing utidelone, particularly in underrepresented populations such as male breast cancer patients, for whom prior clinical trial data on cardiotoxicity are lacking. Further investigation into the potential correlation between UTD1 and cardiac arrhythmias, including prospective studies with sex-specific analyses, is warranted.

## Data Availability

The original contributions presented in the study are included in the article/supplementary material. Further inquiries can be directed to the corresponding author/s.
